# Ivermectin increases striatal cholinergic activity to facilitate dopamine terminal function

**DOI:** 10.1186/s13578-024-01228-2

**Published:** 2024-04-17

**Authors:** Hillary A. Wadsworth, Alicia M. P. Warnecke, Joshua C. Barlow, J. Kayden Robinson, Emma Steimle, Joakim W. Ronström, Pacen E. Williams, Christopher J. Galbraith, Jared Baldridge, Michael W. Jakowec, Daryl L. Davies, Jordan T. Yorgason

**Affiliations:** 1 Department of Cellular Biology and Physiology, and Neuroscience Program, Brigham Young University, 4005 LSB, Provo, UT 84602 USA; 2 Titus Family Department of Clinical Pharmacy, School of Pharmacy, University of Southern California, 1985 Zonal Avenue, Los Angeles, CA 90089 USA; 3 Department of Neurology, Keck School of Medicine, University of Southern California, 1333 San Pablo Street, Los Angeles, CA 90033 USA

**Keywords:** Receptors, Purinergic P2X4 receptors, FSCV, Ivermectin, Parkinson’s

## Abstract

Ivermectin (IVM) is a commonly prescribed antiparasitic treatment with pharmacological effects on invertebrate glutamate ion channels resulting in paralysis and death of invertebrates. However, it can also act as a modulator of some vertebrate ion channels and has shown promise in facilitating L-DOPA treatment in preclinical models of Parkinson’s disease. The pharmacological effects of IVM on dopamine terminal function were tested, focusing on the role of two of IVM’s potential targets: purinergic P2X4 and nicotinic acetylcholine receptors. Ivermectin enhanced electrochemical detection of dorsal striatum dopamine release. Although striatal P2X4 receptors were observed, IVM effects on dopamine release were not blocked by P2X4 receptor inactivation. In contrast, IVM attenuated nicotine effects on dopamine release, and antagonizing nicotinic receptors prevented IVM effects on dopamine release. IVM also enhanced striatal cholinergic interneuron firing. L-DOPA enhances dopamine release by increasing vesicular content. L-DOPA and IVM co-application further enhanced release but resulted in a reduction in the ratio between high and low frequency stimulations, suggesting that IVM is enhancing release largely through changes in terminal excitability and not vesicular content. Thus, IVM is increasing striatal dopamine release through enhanced cholinergic activity on dopamine terminals.

**Supplementary Information:**

The online version contains supplementary material available at 10.1186/s13578-024-01228-2.

## Introduction

Mesostriatal circuitry includes major inputs from dopamine (DA) neurons originating in the substantia nigra compacta (SNc) that synapse onto local medium spiny neurons to modulate output from the direct and indirect striatal pathways. Local regulators of this circuit include large aspiny cholinergic interneurons (CINs) that fire rhythmically due to intrinsic activity [2], and are further activated by glutamate (e.g. thalamic inputs) [36], and by hyperpolarization activated currents driven by GABA inputs [6]. Cholinergic firing can also depolarize DA terminals through activation of nicotinic acetylcholine receptors (nAChRs), resulting in cholinergic-evoked DA release or modulation of ongoing release [7, 45, 58, 68–70, 72]. Alterations in DA inputs and related cholinergic activity are known to drive changes in movement and mood, and underlie symptoms from related disorders such as Parkinson’s disease [1, 8, 39].

The present study examines the interactions between striatal DA and cholinergic local circuits and the commonly prescribed anti-parasitic ivermectin (IVM). IVM is known to affect many DA associated behaviors, including ethanol consumption, anxiety, sensorimotor deficits, and sociocommunicative behavior [19, 28–30, 63, 64]. Indeed, IVM has also been shown to improve L-DOPA induced behaviors, including in preclinical Parkinson’s disease (PD) animal models [30, 59]. However, no studies have been conducted to determine whether IVM influences striatal DA release. Therefore, fast scan cyclic voltammetry (FSCV) was used to measure synaptic DA release in the dorsal striatum (DS) and pharmacological agents were applied during experiments to elucidate the mechanistic effects of IVM on DA release.

## Methods

### Animal subjects

Female and male C57BL/6 (> 30 days-old) were bred and cared for in accordance with the National Institutes of Health Guide for the Care and Use of Laboratory Animals. Animals were housed on a reverse 12:12 h light/dark cycle (lights on from 10 PM to 10 AM) in groups of 2–5/cage and given ad libitum access to food and water. For imaging experiments, Macrophage Fas-Induced Apoptosis (MaFIA) transgenic mice (RRID: IMSR_JAX:005070) were used to image microglia. Experimental protocols were approved by the Brigham Young University Institutional Animal Care and Use Committee according to the National Institutes of Health *Guide for the Care and Use of Laboratory Animals*.

### Brain slice preparation

Coronal brain slices were obtained as previously described [7, 69]. Briefly, animals were anesthetized with isoflurane (5%), decapitated, and brains were rapidly dissected and sectioned into 220 μm slices in artificial cerebrospinal fluid (ACSF) cutting solution. The ACSF cutting solution (pH =  ~ 7.4) was oxygenated at 95% O_2_/5% CO_2_ and consisted of (in mM) 126 NaCl, 2.5 KCl, 1.2 NaH_2_PO_4_, 2.4 CaCl_2_, 1.2 MgCl_2_, 21.4 NaHCO3, 11 glucose and 0.1 ketamine (for glutamate receptor blockade). Slices were transferred to a recording chamber with continuous ACSF flow (2.0 mL/min) maintained at 34–36 °C. The dorsal striatum was visualized at the level of the dorsal horn under low magnification with Nikon Diaphot inverted microscopes in the transmitted light mode and Olympus X51 microscopes with transmitted infrared Dodt gradient contrast imaging.

### Fast scan cyclic voltammetry recordings

Electrically evoked DS DA release was obtained using FSCV. Carbon fiber electrodes (CFEs) were Nafion coated using a 1.5 V 90 s electrodeposition pretreatment for L-DOPA experiments [44]. Dopamine release was electrically evoked every 2 min by monophasic stimulation from a KCl-filled micropipette placed 100–200 μm from the CFE. L-DOPA experiments used an alternating single pulse/5 pulse stimulation protocol (0.5 ms pulse, 350 μA, 20 Hz). Additional experiments used a frequency stimulation protocol that included 5 Hz, 20 Hz, and 100 Hz 5 pulse stimulations. Experiments performed with hexamethonium used a 4 ms pulse to account for the signal disruption with a shorter 0.5 ms pulse. The CFE potential was linearly scanned from − 0.4 to 1.2 V and back to − 0.4 V vs Ag/AgCl (scan rate = 400 V/s). Cyclic voltammograms were recorded every 100 ms (10 Hz) with ChemClamp potentiometers (Dagan Corporation, Minneapolis, MN, USA) or inhouse developed potentiostats. Recordings were performed and analyzed using Demon Voltammetry software as described below [66].

### Drug preparation and administration

IVM (cat. no. NDC 55529-012-01, Norbrook Laboratories, Ltd, Newry, North Ireland, UK), Nicotine (cas. no. 54-11-5, Sigma-Aldrich, St. Louis, Missouri, USA), Hexamethonium bromide (cas. no. 55–97-0, Cayman Chemical Company, Ann Arbor, Michigan, USA), L-DOPA (cat. no. PHR1271, Sigma-Aldrich, St. Louis, Missouri, USA), and 5-(3-Bromophenyl)-1,3-dihydro-2H-benzofuro[3,2-e]-1,4-diazepin-2-one (5-BDBD) (cat. no. T22518, TargetMol, Wellesley Hills, Massachusetts, USA) were dissolved in stock solutions and then diluted into ACSF at specified concentrations (0.1–100 µM IVM, 300 nM Nicotine, 200 µM Hexamethonium, 10 µM L-DOPA, and 10 µM 5-BDBD). Drugs brain slice administration used either gravity-based flow system or peristaltic pumps (1–2 ml/min).

### Multiphoton imaging

A transgenic mouse expressing enhanced green fluorescent protein (EGFP) on the colony stimulating factor 1 receptor on a C57BL/6 J background (Macrophage Fas-Induced Apoptosis, MAFIA) was used to visualize microglia. The animal was anesthetized in 2–4% isoflurane and sacrificed as described above. The brain was extracted, and slices were prepared as described above, targeting the dorsal striatum. The slice was then immediately put into ACSF to incubate with the P2X4 antibody (50:1; Thermo Fisher Scientific, PA5-37,880; AF647 conjugated) at room temperature in the dark for 30 min. The brain slice was moved to the two-photon recording chamber and immersed in ACSF (flow rate of ~ 1–2 ml/min), to wash away excess antibody. The custom in-house built two-photon microscope used a Ti–Sapphire Chameleon Discovery NX Laser (Coherent) tuned to 870 nm (optimized for simultaneous excitement of EGFP and Alexa Fluor 647). Fluorescent emission was visualized using a 40x (0.8NA) water immersion objective (Olympus). A Z-stack was collected with 3 μm between slices, with a total range of 17 slices for simultaneous detection of GFP (520 nm cleanup filter) and Alexa Fluor 647 (670 nm cleanup filter). Images were combined and colorized using Fiji [48].

### Cell-attached electrophysiology recordings in brain slices

Electrophysiology studies utilized borosilicate glass capillary electrodes (2.5–6 MΩ). For cell-attached recordings of CIN firing (NaCl 150 mM inside the pipette), a seal (10MΩ–1 GΩ) was created between the cell membrane and the recording pipette. Spontaneous spike activity was then recorded in voltage-clamp mode with an Axon Instruments Multiclamp 700B (Molecular Devices, San Jose, CA, USA) amplifier and sampled at 3 kHz using an Axon 1440A digitizer (Molecular Devices) and collected and analyzed using Mini Analysis (Synaptosoft: Decatur, GA) and/or Axograph 10 (Axograph, Sydney, Australia). A stable baseline recording of current activity was obtained for 5 min before perfusion of Ivermectin (50 μM) which was applied for ~ 10 min.

### Cholinergic interneuron identification for physiology recordings

The use of firing activity and neuronal size were used to distinguish cholinergic interneurons from other striatal neurons in unlabeled mice using previously identified criteria [5, 26, 31,53, 57, 61, 69, 73]. Cholinergic interneurons are uniformly large and aspiny [31], and spontaneously active [61]. Neurons recorded from were spontaneously active (typically 2–10 Hz under non-drug conditions) and large (> 35 µm). The criterion of being tonically active eliminates the largest GABAergic populations, medium spiny neurons (> 95%; [27, 35]), fast spiking interneurons, neurogliaform and the fast adapting interneurons [52]. Next, the slow firing frequency eliminates the spontaneously active/bursty neurons, since these neurons have a mean firing rate upwards of 100 Hz [52]. By excluding smaller neurons, recordings can also effectively discriminate cholinergic interneurons (20–50 µm; [26]) from other tonically active neurons: low threshold spiking interneurons (neuropeptide Y + /NOS + /somatostatin + ; 10–35 µm; [26] and tyrosine hydroxylase + interneurons (~ 10 µm; [55]). Thus, while the present study is not investigating cholinergic subpopulations, a caveat for the present experiments is that experimenters are biased toward recording only from tonically active neurons > 35 µm in diameter, which may represent an uncategorized subpopulation of cholinergic interneurons.

### Statistical analysis

Dopamine release experiments were analyzed in Demon Voltammetry [66]. To compare across multiple animals and slices, DA release signals were averaged (within subject) across the last three 1 pulse or 5 pulse ACSF recordings to establish a baseline value that subsequent DA signals were normalized to. Thus, post-drug conditions were compared to pre-drug baseline conditions in the same slice for a within-subject experimental design. Electrophysiology CIN firing rate experiments used Axograph and Minianalysis for analysis of action potentials and experiments were also performed using a within-subject design. Statistical analysis was performed using Prism 5 (GraphPad). Significance for all tests was set at *p* < 0.05. Values are expressed as mean ± SEM. Significance levels are indicated on graphs with asterisks *,**,***, corresponding to significance levels p < 0.05, 0.01 and 0.001, respectively. Data will be shared on a per request basis.

## Results

### IVM enhances DA release independent of P2X4 receptor activation

**Fig. 1:**
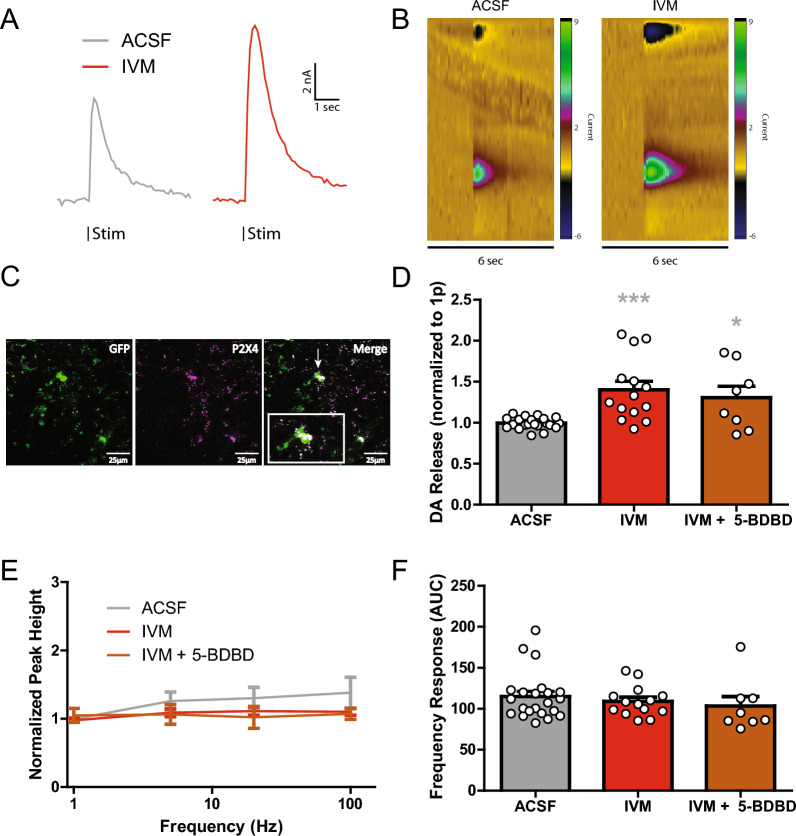
IVM enhances DA release independent of P2X4 receptor activation. **A** Representative traces of single pulse electrical evoked DA release in the DS before (left) and after (right) IVM application. **B** Representative color plots of DA release before (left) and after (right) IVM application. **C** Image showing P2X4 receptor co-localization on microglia in the DS. An antibody against P2X4 receptors was used along with Macrophage Fas-Induced Apoptosis (MaFIA) transgenic mice with GFP labeled microglia. **D** Ivermectin, as well as IVM with 5-BDBD increases single pulse DA release in the dorsal striatum compared to normalized ACSF release. **E** Ivermectin, as well as IVM with 5-BDBD does not affect DA release at multiple frequencies compared to ACSF alone. **F** The overall frequency response, as measured by area under the curve (AUC) of the previous figure **D**, is not affected by IVM or IVM with 5-BDBD. Asterisks *,*** indicate significance levels p < 0.05 and p < 0.001 respectively compared to ACSF pre-treatment

Prior studies have shown that IVM acts as a positive allosteric modulator (PAM) of nAChRs [3, 10] and P2X4 receptors [60], though with greater potency at P2X4 receptors. The concentration dependent effects of IVM on electrically evoked DA release (1 pulse) were examined via bath application (in µM: 0.1, 0.5, 1, 5, 10, 50 and 100) and measured through FSCV (Additional file 1: Fig. S1) one-way ANOVA; F_(7,147)_ = 9.178, p < 0.0001). IVM significantly increased DA release at 50 and 100 µM. The 50 µM concentration was used for the remainder of experiments herein because of clear observed effects that could operate as a positive control in pharmacology experiments. Shown are example traces where IVM (50 µM) resulted in increased electrically evoked DA release (Fig. 1A–B). IVM PAM effects on P2X4 receptors are potent, occurring through the 1–100 µM range [30, 43]. IVM has also been shown to have effects on DA related behaviors through P2X4 receptors [30, 63]. The P2X4 receptor is expressed in the DS, particularly on microglia, but also on non-microglia cells (Fig. 1C). Because of the sensitivity, past behavioral effects, and regional localization, IVM mediated evoked DA release changes were initially hypothesized to occur from P2X4 receptor activation. Co-application of the P2X4 receptor antagonist 5-BDBD (10 µM) did not prevent IVM (50 µM) induced DA increases from single-pulse stimulations (Fig. 1D, one-way ANOVA; *F*_(2,41)_ = 10.32, p = 0.0002). Dopamine release can be sensitive to local circuit activity that manifests as increases in release across faster stimulation frequencies [47, 67, 68]. Therefore, a high to low stimulation frequency protocol was used to examine IVM and P2X4 receptor interactions on DA release circuits. Generally, neither IVM nor 5-BDBD affected the frequency response curve compared to baseline (Fig. 1E; two-way ANOVA; drug, *F*_(2,164)_ = 2.166, p = 0.1179; frequency, *F*_(3,164)_ = 0.7402, p = 0.5295; interaction, *F*_(6,164)_ = 0.3469, p = 0.9109). The frequency response area under the curve (AUC) remained unchanged between the different treatments (Fig. 1F; one-way ANOVA; *F*_(2,41)_ = 0.5950, p = 0.5563). Together, this data indicates that IVM-induced increases in DS DA release are independent of P2X4 receptor activation and that IVM has no apparent effect on the DA frequency response.

### IVM attenuates nicotine effects on DA release

**Fig. 2:**
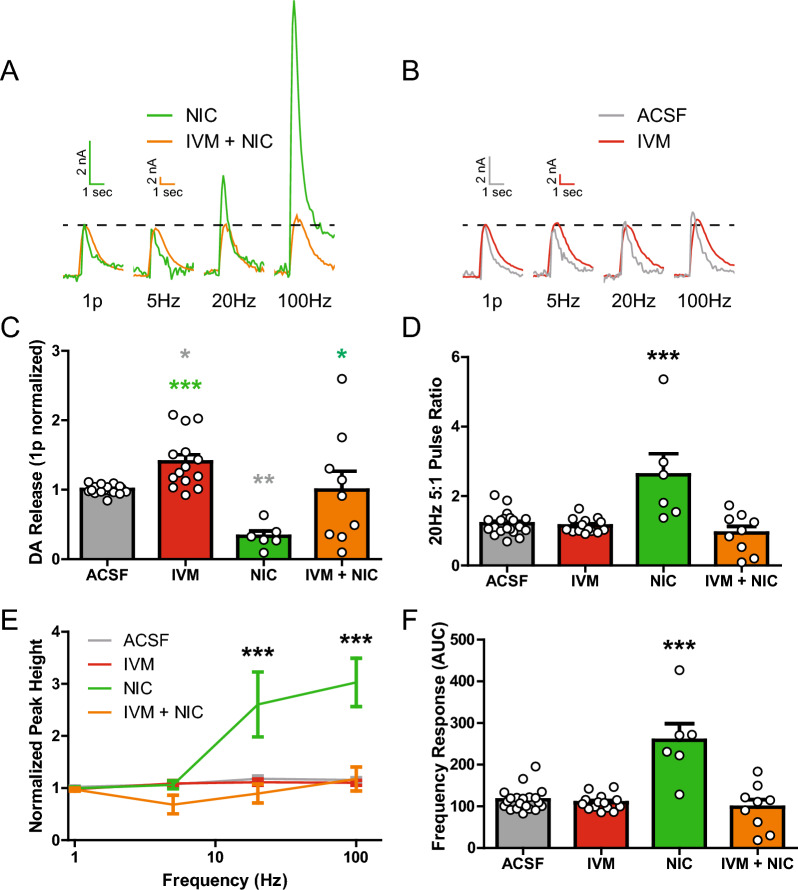
IVM attenuates nicotine effects on DA release. **A** Representative traces of electrically evoked DA release in the DS at multiple frequencies. Comparing nicotine (NIC) and IVM + NIC, each trace is normalized to 1 pulse release (dotted line). **B** Representative traces of DS DA release at multiple frequencies comparing ACSF and IVM. Each trace is normalized to 1 pulse release (dotted line). **C** Ivermectin increases single pulse DA release while NIC decreases DA release. Ivermectin attenuates the effect of NIC on single pulse dopamine release. **D** Nicotine alone increases the 20 Hz 5 pulse ratio compared to ACSF, IVM, and IVM + NIC. **E** Nicotine alone shows increased DA release in response to high frequency, 5 pulse stimulations, with AUC represented in **F**. Asterisks *,**(grey) indicate significance levels p < 0.05 and p < 0.01 respectively compared to ACSF pre-treatment. Asterisks *,***(green) indicate significance levels p < 0.05 and p < 0.001 respectively compared to nicotine treatment. Asterisks ***(black) indicate significance levels p < 0.001 compared to all other treatments

IVM is a known nAChR PAM [10, 34] and nAChRs are powerful activators and modulators of DA terminal activity [69]. Further, nAChRs PAMs, such as alcohol, increase DA terminal function resulting in greater release [20]. Accordingly, experiments were conducted to assess if IVM modifies known nAChR effects on DA release. Cholinergic interneurons release acetylcholine to directly depolarize DA terminals [58, 72]. Under conditions where nAChRs are lightly stimulated, such as in the presence of low concentrations of acetylcholinesterase inhibitors, single pulse evoked DA release is enhanced [71, 72]. However, prolonged activation of nicotinic receptors (such as the presence of a high concentration of the full agonist nicotine) results in receptor desensitization [23], and subsequent alterations in electrically evoked DA release which are often described as a high-pass filter, and release becomes biased towards high frequency stimulation conditions [45, 70, 72] (Fig. 2A). IVM itself did not strongly exhibit high-pass filter properties (Fig. 2B). Next, experiments were conducted to test if IVM influenced nicotine’s effects on DA release (Fig. 2). In contrast to IVM facilitatory effects on single pulse stimulated DA release, nicotine alone reduced DA release likely due to desensitization of the nAChRs [72]. However, concurrent application of IVM (50 µM) and nicotine (300 nM) resulted in increased variability and significantly impaired nicotine inhibitory effects on DA release (Fig. 2C; one-way ANOVA; *F*_(3,45)_ = 9.944, p < 0.0001). Furthermore, nicotine alone enhanced the 20 Hz 5:1 pulse ratio, indicating the typical high-pass filter effect (Fig. 2D). However, IVM and nicotine co-application resulted in a reduction in this ratio similar to baseline and IVM alone (Fig. 2D; one-way ANOVA; *F*_(3,45)_ = 11.11, p < 0.0001). Examining the full frequency stimulation condition curve, the well characterized high-pass filter effect was observed with nicotine (Fig. 2A, E). However, this effect was greatly diminished by IVM co-application, and IVM alone had no apparent effect on the frequency response (Fig. 2A–B,E; two-way ANOVA; drug, *F*_(3,209)_ = 30.97, p < 0.0001; frequency, *F*_(3,209)_ = 25.48, p < 0.0001; interaction, *F*_(9,209)_ = 10.37, p < 0.0001). IVM appears to block or counter the effects of nicotine on DA release from single pulse and multiple pulse high frequency stimulations, suggesting that IVM may be preventing nAChR desensitization through allosteric effects. This finding is summarized in the AUC measures from the stimulation frequency response curve (Fig. 2F). The inclusion of IVM with nicotine significantly reduced the AUC magnitude compared to nicotine alone (Fig. 2F; one-way ANOVA; *F*_(3,45)_ = 19.93, p < 0.0001). Thus, IVM enhances DA release on its own and opposes the nicotine-induced nAChR desensitization and high-pass frequency bias, suggesting that IVM effects on DA release may involve effects on the acetylcholine system.

### IVM attenuation of nicotine effects is not through P2X4 receptors

**Fig. 3:**
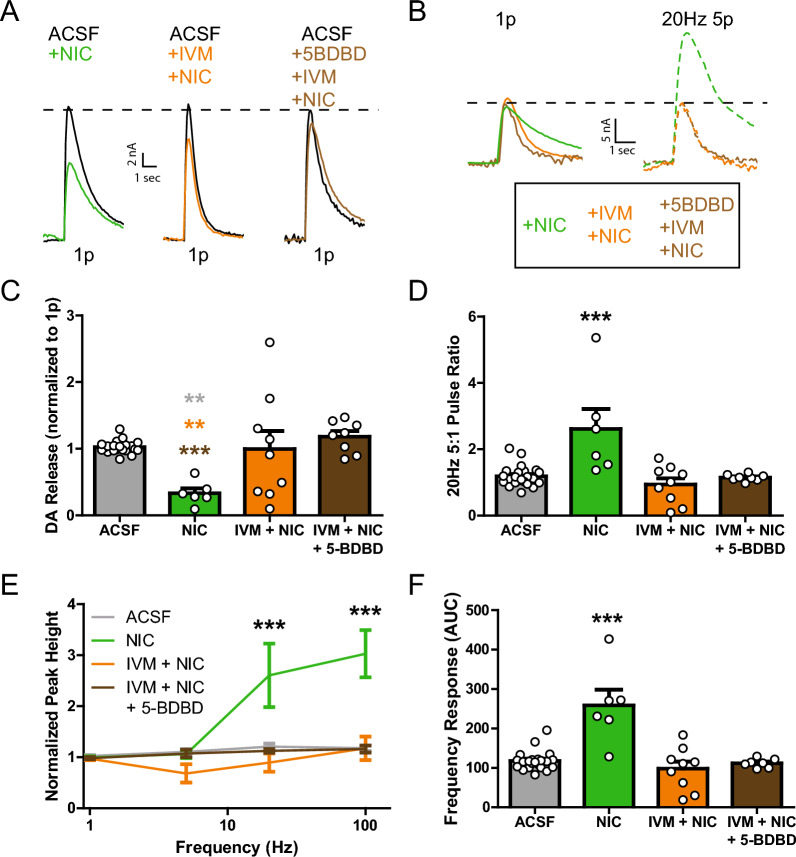
IVM attenuation of nicotine effects is not through P2X4 receptors. **A** Representative traces of single pulse electrically evoked DA release in the DS. Nicotine (NIC) alone decreases single pulse DA release. **B** Representative traces of evoked DA release at multiple frequencies. Nicotine alone increases release with 20 Hz 5 pulse stimulation compared to co-application with IVM or IVM and 5-BDBD. **C** Nicotine decreases single pulse DA release while IVM + NIC, and IVM + NIC + 5-BDBD have no effect on single pulse DA release compared to ACSF pre-treatment. **D** Nicotine alone increases the 20 Hz 5 pulse ratio compared to ACSF, IVM + NIC, and IVM + NIC + 5-BDBD. **E** Nicotine alone shows increased DA release in response to high frequency, 5 pulse stimulations, with AUC represented in **F**. Asterisks **(grey) indicate significance levels p < 0.01 compared to ACSF pre-treatment. Asterisks **(orange) indicate significance levels p < 0.01 compared to IVM + NIC treatment. Asterisks ***(brown) indicate significance levels p < 0.001 compared to IVM + NIC + 5-BDBD. Asterisks ***(black) indicate significance levels p < 0.001 compared to all other treatments

IVM-mediated DA enhancement is not through IVM-PAM effects on P2X4 receptors. However, prior work has indicated that P2X4 receptors are necessary for IVM’s behavioral effects [30, 62]. Further, since several subpopulations of the striatum express nicotinic receptors, additional DS circuitry may be recruited in the presence of nicotine to influence local circuit activity and could influence P2X4 receptor activity. Thus, nicotine experiments were also performed in the presence of 5-BDBD to rule out a possible interaction (Fig. 3). If IVM is modulating the nicotinic effect through P2X4 receptor interactions, then presence of the P2X4 antagonist 5-BDBD should restore nicotinic effects on DA release. Experiments with IVM, nicotine, and 5-BDBD applied simultaneously revealed a similar effect to ACSF alone and was significantly different from nicotine alone (Fig. 3A,C; one-way ANOVA; *F*_(3,42)_ = 6.779, p = 0.0008). Similarly, nicotine effects on the 5:1 pulse ratio were not restored with 5-BDBD application (Fig. 3B,D; one-way ANOVA; *F*_(3,42)_ = 10.64, p < 0.0001). 5-BDBD did not restore the nicotine high frequency DA release effects (Fig. 3E; two-way ANOVA; drug, *F*_(3,163)_ = 24.71, p < 0.0001; frequency, *F*_(3,163)_ = 18.82, p < 0.0001; interaction, *F*_(9,163)_ = 8.085, p < 0.0001), which is summarized with the AUC measures from the frequency response (Fig. 3F; one-way ANOVA; *F*_(3,40)_ = 17.98, p < 0.0001). Therefore, IVM effects on the cholinergic system are independent of P2X4 receptor activation.

### IVM enhances striatal cholinergic interneuron firing activity

**Fig. 4:**
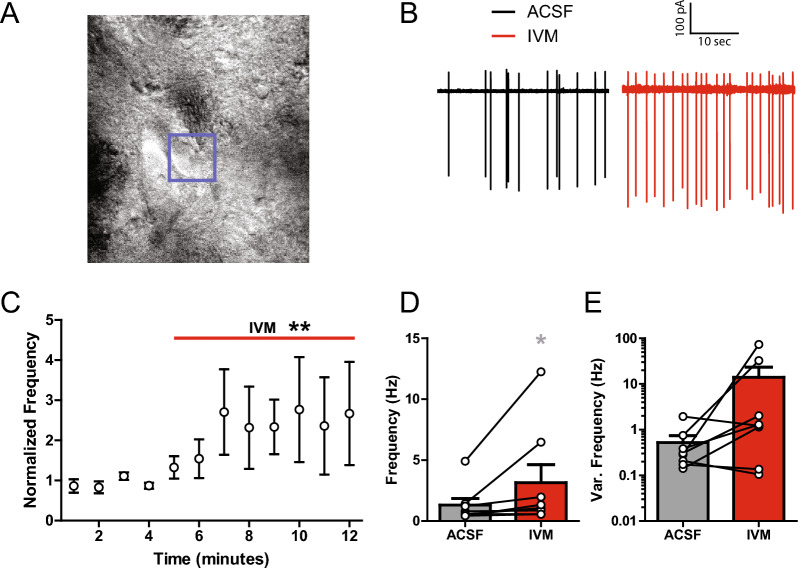
IVM effects on striatal cholinergic interneurons. **A** Representative image of glass capillary electrode connected to a DS CIN. **B** Representative traces of CIN firing rate before (left) and after IVM application (right). **C** Time course of CIN normalized frequency showing IVM is increasing DS CIN firing rate. **D** Ivermectin increases CIN firing rate frequency. **E** Action potential frequency variance (Var.) was increased in some but not all neurons, and group changes were not statistically significant. Asterisks *,** indicate significance levels p < 0.05, p < 0.01 compared to ACSF pre-treatment

Although IVM is known to interact with nicotinic receptors [10, 34], IVM acetylcholine interactive effects may also involve indirect effects on CINs, and subsequent changes in acetylcholine release. Therefore, the effects of IVM on DS CIN firing was examined using cell-attached electrophysiology experiments. CINs were visually identified and patched onto using borosilicate glass capillary electrodes (Fig. 4A). IVM increased the CIN firing rate frequency from 1.32 ± 0.53 to 3.15 ± 1.47 Hz (Fig. 4B–C,D; Two-tailed Wilcoxon Matched Pairs test; *p* = 0.0156). The variance of firing frequency trended toward increases but was not significant (Fig. 4E; Two-tailed Wilcoxon Matched Pairs test; *p* = 0.1094). Thus, some of IVM effects are through excitatory effects on CIN firing, which are likely influencing the nicotine response observed in FSCV studies. Regardless, these data support the hypothesis that IVM is affecting DA release and nicotine effects through its effects on the striatal acetylcholine system.

### IVM induced increases in DA release is through nAChRs

**Fig. 5:**
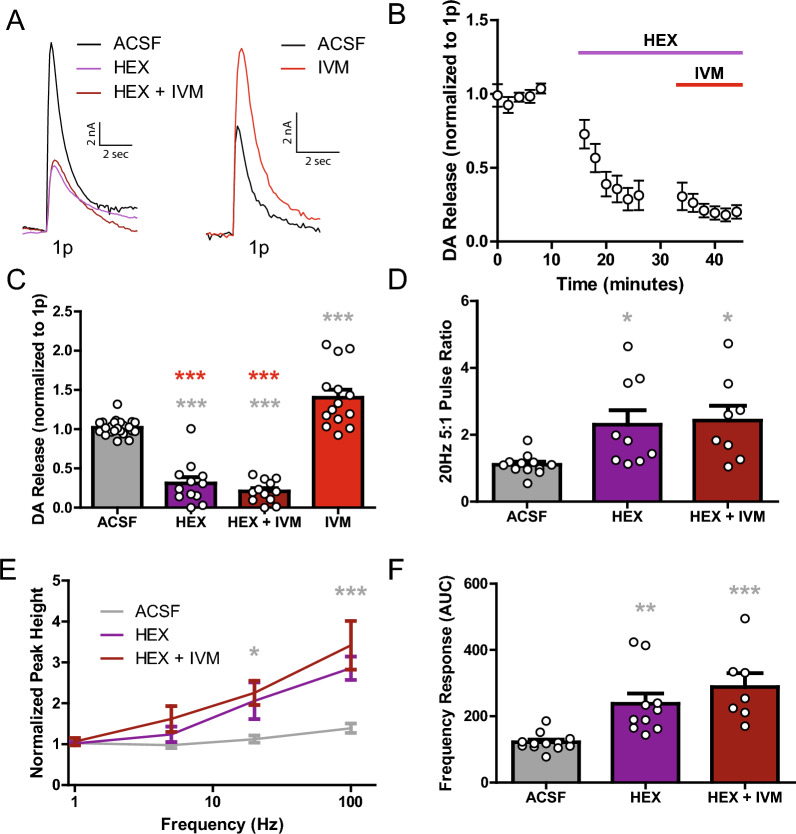
IVM effect on DA release is through nAChRs. **A** Representative traces of single pulse electrical evoked DA release in the DS with HEX (purple) and HEX + IVM (maroon) compared to ACSF pre-treatment (black). Separate experiments showed the IVM increases DA release (ACSF: black, IVM: red). **B** Time course of single pulse DA release showing HEX induced decreases in DA release which is not rescued by concurrent IVM application. **C** Hexamethonium, and HEX + IVM decrease single pulse DA release compared to pre-treatment ACSF levels and to IVM, which increases single pulse DA release. **D** Hexamethonium and HEX + IVM increase the 20 Hz 5 pulse ratio compared to ACSF pre-treatment. **E** Both HEX and HEX + IVM show increased DA release in response to high frequency, 5 pulse stimulations compared to ACSF pre-treatment. **F** Overall frequency response as measured by AUC. Asterisks *,**,***(grey) indicate significance levels p < 0.05, p < 0.01, and p < 0.001, respectively compared to ACSF pre-treatment. Asterisks ***(red) indicate significance levels p < 0.001 compared to IVM

Whether IVM has its dopaminergic effects through nAChRs was explored next. The nAChR antagonist hexamethonium (HEX; 200 µM) was applied to brain slices and interactions with IVM effects on DA release measured. Single pulse DA release was greatly reduced by HEX (Fig. 5A–C). When IVM was added to HEX, no noticeable change occurred for single pulse DA release, and normalized signals remained significantly lower compared to both baseline and IVM alone (Fig. 5A–C; one-way ANOVA; *F*_(3,60)_ = 84.14, p < 0.0001). Qualitatively, similar to that observed with nicotine, the 5:1 pulse ratio (20 Hz) was increased after HEX, with no further increase observed with co-application of IVM + HEX (Fig. 5D; one-way ANOVA; *F*_(2,26)_ = 5.688, p = 0.0089). The frequency response for HEX and HEX + IVM were both significantly larger than pre-drug conditions, with normalized peak height becoming increasingly significant at higher frequencies (Fig. 5E; two-way ANOVA; drug, *F*_(2,117)_ = 14.85, p < 0.0001; frequency, *F*_(3,117)_ = 21.06, p < 0.0001; interaction, *F*_(3,117)_ = 3.229, p = 0.0057). In measuring the overall frequency response of each treatment using AUC, both HEX alone and HEX + IVM significantly increased the frequency effect compared to baseline levels (Fig. 5F; one-way ANOVA; *F*_(2,26)_ = 10.89, p = 0.0004). Together with agonist data from Fig. 2, the results in Figs. 4, 5 support the hypothesis that IVM increases DA release through enhanced nAChR activity.

### IVM increases DA release with L-DOPA

**Fig. 6:**
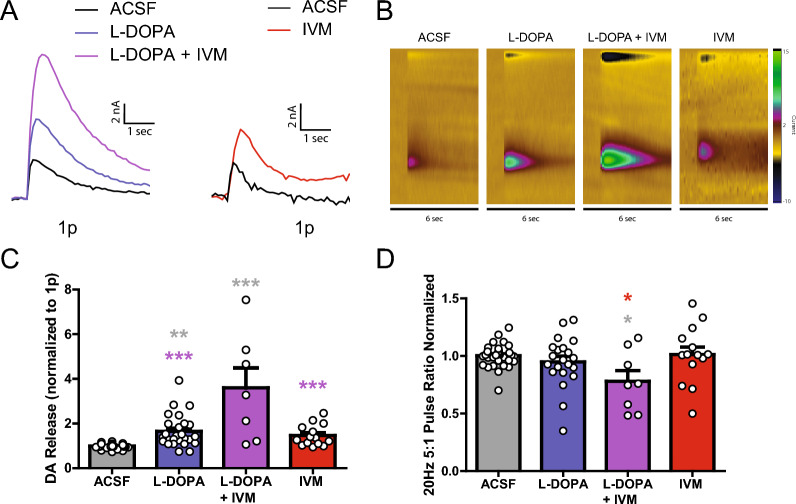
IVM increases DA release with L-DOPA. **A** Representative traces of single pulse electrical evoked DA release in the DS comparing pre-treatment ACSF (black), L-DOPA (blue) and L-DOPA + IVM (purple). Separate experiments showed the IVM increases DA release (ACSF: black, IVM: red). **B** Representative color plots of DA release with pre-treatment ACSF (first), L-DOPA (second), L-DOPA + IVM (third), and IVM (fourth). **C** L-DOPA and L-DOPA + IVM significantly increases single pulse DA release compared to ACSF pre-treatment levels. **D** L-DOPA + IVM significantly decreases the normalized 20 Hz 5 pulse ratio compared to ACSF pre-treatment, while L-DOPA alone and IVM alone have no effect. Asterisks *,**,***(grey) indicate significance levels p < 0.05, p < 0.01, and p < 0.001, respectively compared to ACSF pre-treatment. Asterisks ***(purple) indicate significance levels p < 0.001 compared to L-DOPA + IVM. Asterisks *(red) indicate significance levels p < 0.05 compared to IVM

Prior work has shown that IVM enhances L-DOPA induced behaviors [59]. In consideration of this IVM and L-DOPA interaction, experiments were performed to test if IVM enhances L-DOPA effects on DA release. Application of L-DOPA increases single pulse DA release, which was significantly increased further with co-application of L-DOPA and IVM (Fig. 6A–C; one-way ANOVA; *F*_(3,83)_ = 22.74, p < 0.0001). In separate experiments, IVM increased DA release (Fig. 6A–C). Application of L-DOPA or IVM alone had no effect on the normalized 5:1 pulse ratio (20 Hz) of DA release. However, combined application of L-DOPA with IVM statistically decreased the 5:1 pulse ratio compared to baseline levels (Fig. 6D; one-way ANOVA; *F*_(3,74)_ = 3.486, p = 0.0199). These results are reflective of paired pulse stimulation studies where enhanced release during the primary stimulation results in an attenuation in a secondary pulse [38, 46]. Therefore, the reduced 5:1 pulse ratio with IVM + L-DOPA co-application is likely due to increased release probability during the 1 pulse stimulation resulting in reduced readily releasable pools in the 5 pulse stimulation condition. This suggests that IVM increases DA release past what L-DOPA does alone, and that IVM effects are unique in mechanism to that of L-DOPA.

## Discussion

The present goal was to determine the effects of IVM on striatal DA release and pharmacological mechanisms. Ivermectin increased striatal DA release independent from P2X4 receptor activation. Furthermore, IVM reduced nicotine desensitization effects on DA release, and IVM effects were blocked by nicotinic receptor antagonism. Since IVM also enhanced CIN firing, IVM is likely influencing DA release through multiple cholinergic related mechanisms. Importantly, when IVM was co-applied with L-DOPA, there was a greater amount of DA release than with L-DOPA alone. L-DOPA is the biosynthetic precursor to DA and increases DA release by increasing the DA vesicular content [41]. Ivermectin increases single pulse stimulation-mediated DA release but decreases the high frequency to low frequency DA release ratio when combined with L-DOPA. This decrease in ratio indicates that IVM effects are mostly through DA terminal excitation and not through additional changes in vesicular content.

Ivermectin has FDA approval and is used clinically to treat tropical parasitic diseases [18, 42]. While IVM was originally used for veterinary medicine in 1981, it was later approved for use in humans in 1988 [13], specifically for onchocerciasis, helminthiases and scabies [42]. It is the only recommended oral medication for scabies [22]. Ivermectin works as an anti-parasitic by binding to invertebrate glutamate-gated chloride channels, causing hyperpolarization of parasite neurons and muscles leading to paralysis and eventually death [21]. Ivermectin is also anti-inflammatory and relatively well tolerated [32]. Because IVM is relatively safe, FDA approved, and commonly prescribed, it is important to understand its effects on neural circuitry. Furthermore, because it is an allosteric modulator, it has potential for amplifying pharmacological effects of other substances used clinically. Ivermectin itself increases DA release in the DS through cholinergic mechanisms, which could potentially benefit those with dysfunction in DA circuity, including those with PD, mood disorders, or attention deficit disorder.

### IVM effects through acetylcholine related mechanisms

Throughout the dorsal and ventral striatum, CINs are important frequency dependent modulators of DA release [17, 40] and can drive DA release independent of DA cell firing [58, 69]. Drugs that modulate CIN activity and nicotinic acetylcholine receptors on DA terminals enhance the ratio of DA release from high to low frequency stimulations, a well-characterized effect described as a high-pass filter [45, 51, 70, 72]. Despite the powerful regulation of DA by CINs, the present study did not indicate any potentiation of DA release under high frequency stimulations in the presence of IVM alone. This contrasted with IVM attenuating the nicotine-induced changes in DA release. This demonstrates that IVM is affecting the DS cholinergic system to modulate DA release. Since IVM directly increases CIN firing frequency, increases in DA release likely involve this change in CIN excitability and subsequent downstream effects on nAChRs. These findings were further verified by the application of HEX, which blocks CIN input onto DA terminals. By inhibiting the effects of acetylcholine through nAChR antagonism, IVM no longer increased single pulse DA release. Thus, IVM is likely acting through nAChRs on DA terminals to enhance release. Interestingly, in the presence of IVM, HEX still induced a high-pass filter effect for multiple pulse stimulations. This suggests that nAChRs are the final common pathway for IVM effects, further strengthening the hypothesis that IVM is working through the cholinergic system and the communication between CIN and DA terminals.

It may be surprising that nAChR activity could enhance DA release, since nicotine reduced DA release here and previously [47, 72]. However, it should be noted that this reduction in evoked DA release is likely due to receptor desensitization, and that a milder nAChR stimulation would result in enhanced DA release. For instance, channel rhodopsin mediated stimulation of cholinergic interneurons results in DA release [58] that is nAChR dependent [54]. Further, cholinesterase inhibitors (ambenonium, galantamine and donepezil), which function to enhance acetylcholine levels [37], increase evoked DA release at low but inhibit at high concentrations [70]. Similarly, we have also observed previously clear PAM effects of ethanol on nAChRs that resulted in DA terminal enhancement at low concentrations [20], but inhibition at high concentrations [67]. Both excitatory and inhibitory ethanol effects are blocked by nAChR antagonists in these prior studies. Further support for nAChR desensitization at high concentrations comes from recent patch clamp studies that observed clear reductions in nAChR currents with prolonged cholinesterase inhibitor application [33]. Thus, we speculate that IVM effects for enhancing DA release are through impaired nAChR desensitization, resulting in greater DA terminal excitation and subsequent release.

### IVM effects on P2X4 receptors

Ivermectin is a PAM on P2X4 receptors, nicotinic receptors and γ-aminobutyric acid type A (GABA_A_) receptors [4, 9, 12, 14, 16, 25, 30, 62, 64]. Notably, in P2X4 receptor knockout (KO) mice, the behavioral effects of IVM are diminished. Specifically, IVM decreases ethanol drinking and increases motor behavior, and these IVM effects are P2X4 receptor mediated [30, 62].

Based on these prior studies, IVM effects on DA release were hypothesized to include the P2X4 receptors. However, multiple experiments using P2X4 receptor antagonist 5-BDBD were performed, and P2X4 antagonism did not block any of IVM’s effects on DA release, including nicotine interactions. However, it’s possible that P2X4 receptors are involved in other aspects of these circuitry not tested herein. For instance, the P2X4 receptor is expressed on immune cells in the periphery, as well as microglia in the brain, which are activated through peripheral cytokine networks [24]. Furthermore, P2X4 receptor involvement could be present in other brain regions, including upstream circuits not present in brain slices. For instance, all present experiments were performed in DS brain slices, which is associated with motor behavior and decreases in DA release with PD[50]. Therefore, the previous behavioral P2X4 KO studies have a peripheral immune component and additional circuitry components that were presently excluded. Previous work connecting P2X4 receptors to motor behaviors using P2X4 KO mice demonstrate that P2X4 receptors are involved in regulating DA terminal function [30]. Considering present experiments were only looking at acute IVM and 5-BDBD exposure, there may be effects of P2X4 receptors on DA release more directly over prolonged exposure. Regardless, current results provide strong evidence for IVM effects on DA terminals that are dependent on local cholinergic systems. It is important to note that IVM may also have effects on other ligand gated ion channels, including glutamate-gated chloride channels and glycine receptors [15, 49], though these additional receptors were not examined here.

### L-DOPA

Previous studies in a mouse PD model showed that L-DOPA with IVM could alter DA motor behavior to a greater extent than L-DOPA on its own [30, 59]. Presently, L-DOPA application increased DA release, which increased further with IVM co-application. The results of these experiments suggest that the behavioral effects seen in prior PD mouse models are due to L-DOPA and IVM’s ability to increase DA release through multiple mechanisms, specifically, increased vesicle DA content with L-DOPA [44], and increased release probability with IVM. Multimodal treatment approaches are common for PD [56]. Ivermectin and L-DOPA interactions in humans have not been reported to date, though considering IVM and L-DOPA are both commonly prescribed, the lack of published studies suggests mild interactions at most for prescribed doses. Regardless, the present experiments suggest an interaction that may facilitate and/or complicate treatment. This was not further explored; L-DOPA was used as an experimental variable to test for mechanistic effects of IVM, and not to inform on PD treatment.

From L-DOPA experiments, it was noteworthy that while neither IVM nor L-DOPA alone affected the 5:1 pulse ratio (both drugs enhanced release, but enhanced release similarly across stimulation conditions), co-application of IVM + L-DOPA saw a decrease in the 5:1 pulse ratio (high frequency stimulations had reduced effects on release). Since the release from 1 pulse experiments was greater in the combined drug experiments, this suggests that the ratio reduction is due to a reduced readily releasable pool of DA in IVM + L-DOPA conditions. This is similar to prior electrophysiology studies where a paired pulse stimulation shows decreased release for second pulse stimulation compared to the initial pulse stimulation [38, 46]. It has been shown previously that repeated electrical stimulations reduces the readily releasable DA pool [65]. A similar change in release probability has been shown with methamphetamine in vivo models, where readily releasable pools increased while reserve pools are depleted, showing changes in DA release dependent on stimulation type [11]. L-DOPA is precursor to DA and it works primarily by increasing the readily available DA. This reduced 5:1 pulse ratio with IVM + L-DOPA co-application suggests that IVM increases DA release past what L-DOPA does alone, and that IVM effects are unique in mechanism to that of L-DOPA, because this co-application shows a decrease in the readily-releasable pools of DA release for multi-pulse stimulations.

## Conclusion

This study provides novel information about the effects of IVM on DA release *in slice*. Dopamine release in the striatum is mediated by many intrinsic factors, including ion channels, autoreceptors and heteroreceptors. Through FSCV experiments, IVM is increasing DA release independent of P2X4 receptor activity in the DS, even though P2X4 receptors are in this region on microglia as well as other cells. Ivermectin also attenuates nicotine effects as a high-pass filter in a way that is not mediated through P2X4 receptors. In addition, IVM is able to increase CIN firing rate frequency, highlighting a possible mechanism through which IVM is acting to affect DA release. With that, HEX was used to block CIN outputs on nAChRs and saw that IVM no longer was able to increase DA release. Ivermectin was also unable to block HEX as a high-pass filter, suggesting that IVM is affecting DA release directly through nAChRs, providing further evidence of IVM acting on DA release through CINs. And when IVM was co-applied with L-DOPA there was an even greater increase of DA release compared to IVM or L-DOPA alone. In addition, co-application of L-DOPA with IVM saw a decrease in the normalized 5 pulse ratio, showing that L-DOPA and IVM together is decreasing the readily releasable pool of DA, meaning that IVM is acting through a different mechanism then L-DOPA. This study has helped elucidate the effects of IVM on DA release and how it is able to mediate the cholinergic system in the DS.

### Supplementary Information

**Additional file 1: Figure. S1.** IVM Dose Response Curve. IVM effects on dopamine release in the dorsal striatum were tested at various concentrations (in µM; 0.1, 0.5, 1, 5, 10, 50 and 100). Only 50 µM and 100 µM IVM statistically increased single pulse dopamine release in the dorsal striatum compared to normalized ACSF release. Asterisks *** indicate significance levels p < 0.001 compared to ACSF pre-treatment.

## Data Availability

The dataset generated during and/or analyzed during the current study will be made available upon request.
