# Supplementary figures and images for: Ivermectin increases striatal cholinergic activity to facilitate dopamine terminal function
Source: Cell Biosci. 2024 Apr 17;14:50. doi: 10.1186/s13578-024-01228-2 (PMC11025261; doi:10.1186/s13578-024-01228-2)

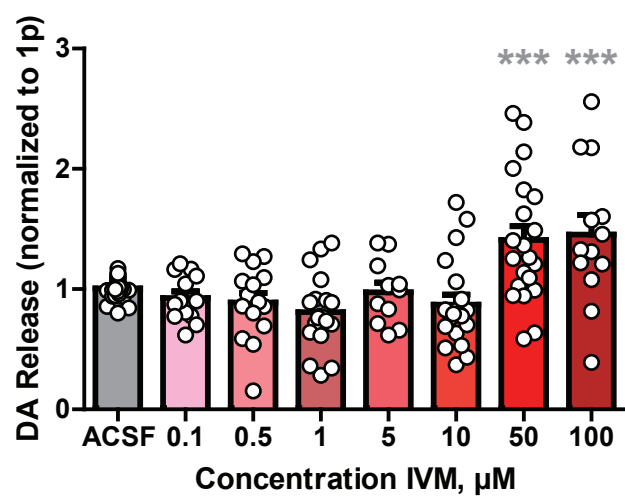

Sup. Fig. IVM Dose Response Curve

Supplement: Supplementary file 1 — Additional file 1: Figure. S1. IVM Dose Response Curve. IVM effects on dopamine release in the dorsal striatum were tested at various concentrations (in µM; 0.1, 0.5, 1, 5, 10, 50 and 100). Only 50 µM and 100 µM IVM statistically increased single pulse dopamine release in the dorsal striatum compared to normalized ACSF release. Asterisks *** indicate significance levels p < 0.001 compared to ACSF pre-treatment. [file 13578_2024_1228_MOESM1_ESM.pdf]
